# Orbital tuberculosis mimicking a vascular emergency: A case report of superior ophthalmic vein engorgement

**DOI:** 10.1016/j.idcr.2026.e02534

**Published:** 2026-02-22

**Authors:** Mansoor Shahriari, Mohsen Zare, Amirhossein Moghtader Mojdehi

**Affiliations:** a Department of Ophthalmology, Imam Hossein Educational Hospital, School of Medicine, Shahid Beheshti University of Medical Sciences, Tehran, Iran; b Department of Infectious Diseases, Imam Hossein Educational Hospital, School of Medicine, Shahid Beheshti University of Medical Sciences, Tehran, Iran

**Keywords:** Orbital tuberculosis, Superior ophthalmic vein engorgement, Carotid–cavernous fistula, Case report

## Abstract

We report a rare case of orbital tuberculosis (O-TB) in an elderly diabetic patient that mimicked a variety of vascular, infectious, and inflammatory orbital diseases. A 73-year-old woman with uncontrolled diabetes, unilateral swelling of the left eye, proptosis, and binocular diplopia, as well as left superior ophthalmic vein (SOV) engorgement on an orbital CT scan, was referred to our center to rule out a carotid-cavernous fistula (CCF) or a cavernous sinus thrombosis (CST). An MRI, MRV, and cerebral angiography ruled out both diagnoses. Nasal endoscopy revealed septal necrosis, and biopsy demonstrated infectious necrosis. Initial bacterial and fungal smear and cultures were negative. However, Ziehl–Neelsen staining and culture on Lowenstein–Jensen medium were positive for acid-fast bacilli, and PCR confirmed *Mycobacterium tuberculosis*. The patient was treated with anti-tuberculosis treatment and recovered two months after treatment. O-TB may rarely mimic a vascular emergency through isolated superior ophthalmic vein engorgement. Clinicians should specifically consider orbital tuberculosis in patients with orbital inflammation and vascular-mimicking imaging findings when routine vascular evaluations are inconclusive, particularly in tuberculosis-endemic regions, to avoid diagnostic delay and ensure timely and appropriate treatment.

## Introduction

Orbital tuberculosis (O-TB) is a rare manifestation of extrapulmonary tuberculosis (TB) that may occur with or without concomitant pulmonary disease [1]. Although most reports have come from endemic areas, there have been reports of an increased incidence of extrapulmonary TB in non-endemic developed countries due to immigration and the prevalence of immunodeficiency [2]. Because of its rarity and nonspecific symptoms, diagnosing it is a serious challenge for clinicians. Delaying diagnosis or prematurely prescribing corticosteroids can lead to irreversible complications for patients [3]. Patients may present with symptoms, such as proptosis, eyelid swelling, pain, diplopia, and decreased vision, that overlap with those of many orbital diseases [3]. Imaging findings are essential for assessing the severity of orbital involvement. However, definitive diagnosis requires microbiological or histopathological confirmation through acid-fast bacilli (AFB) staining or polymerase chain reaction (PCR) for *Mycobacterium tuberculosis* [4]. Despite these challenges, a timely diagnosis can lead to a favorable prognosis [5].

We present an unusual case of O-TB in an elderly patient with uncontrolled diabetes. Due to the patient's characteristics, clinical presentation, and imaging findings, the case mimicked various vascular, infectious, and inflammatory orbital diseases. Ultimately, the diagnosis was established following nasal endoscopy with biopsy, which showed infectious necrosis, and was subsequently confirmed by positive Ziehl–Neelsen staining, culture, and PCR for *Mycobacterium tuberculosis*.

## Case presentation

On September 14, 2024, a 73-year-old woman presenting with binocular diplopia and proptosis was referred to the Orbit Clinic at Imam Hossein Hospital in Tehran. Five days after the onset of upper respiratory symptoms, including rhinorrhea, she began experiencing proptosis, swelling around the left eye, and diplopia. At that time, the patient did not mention decreased vision but reported vague pain during eye movements. Due to the progression of her symptoms, she consulted a doctor, who prescribed ceftriaxone and hydrocortisone. This resulted in an improvement in swelling, but her diplopia continued. She was then referred to a tertiary center. After an orbital CT scan revealed superior ophthalmic vein (SOV) engorgement in the left eye, she was referred to our hospital for a neurological evaluation due to the suspicion of carotid-cavernous fistula (CCF) or a cavernous sinus thrombosis (CST). Cerebral angiography showed no evidence of CCF, and Magnetic Resonance Imaging (MRI) and Magnetic Resonance Venography (MRV) ruled out CST. The patient was finally referred to our service for further evaluation.

She had a history of poorly controlled diabetes mellitus and systemic hypertension but no history of trauma, insect bites, malignancy, or systemic disease.

**Fig. 1:**
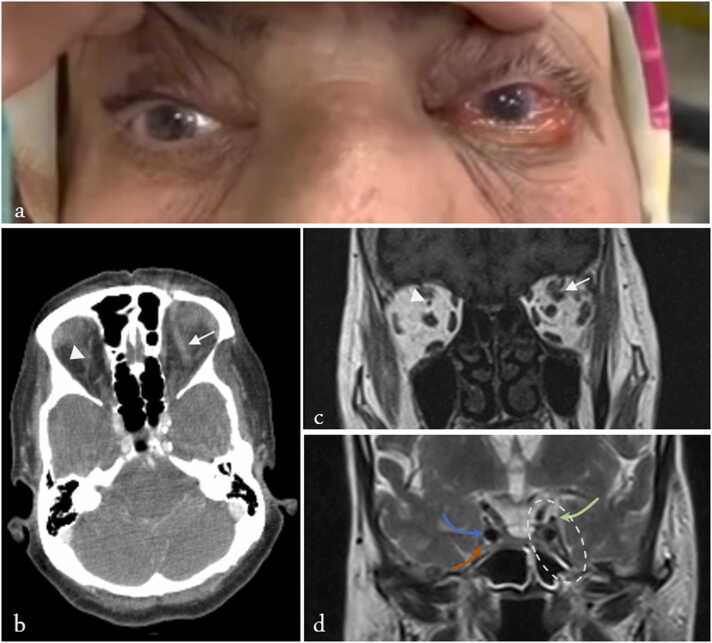
Clinical and radiologic findings of the patient at presentation. (A) External photograph of the patient showing left-sided proptosis and chemosis. (B) Axial CT scan demonstrating left superior ophthalmic vein (SOV) engorgement (arrow) and normal right SOV (arrowhead). (C) Coronal T1-weighted MRI showing dilatation of the left SOV (arrow) and normal right SOV (arrowhead). (D) Coronal T2-weighted MRI illustrating the cavernous sinus region. The left cavernous sinus is outlined with a white dashed oval. The oculomotor nerve (cranial nerve III), the internal carotid artery, and the V2 branch of the trigeminal nerve are indicated with green, blue, and orange arrows, respectively.

Best-corrected visual acuity (BCVA) was 6/10 in the right eye and 5/10 in the left. Relative afferent pupillary defect (RAPD) was negative. Due to illiteracy, color vision could not be tested with the Ishihara plate. An external examination of the left eye revealed mild periorbital swelling, 3 + limitation of movement in all directions, and non-pulsatile axial proptosis (Hertel exophthalmometer: almost 2 mm proptosis). There was no ptosis, redness, warmth, skin ulceration, or abnormality in the sensory examination of the trigeminal nerve distribution (See Fig. 1.).

Slit lamp examination revealed unilateral 4 + chemosis, no episcleral corkscrew vessels, and bilateral moderate nuclear cataracts. Intraocular pressure (IOP) was within the normal range and symmetrical. The optic nerve head appeared normal, and there was no evidence of diabetic retinopathy. An oral examination revealed no evidence of dental caries or oral ulcers.

At this stage, we reviewed the previous medical team's findings. Points of interest, some of which were previously mentioned, included left SOV engorgement in orbital CT and MRI, as well as retrobulbar fat enhancement (Fig. 1, Supplementary Fig. 1). Based on the patient's clinical presentation, we considered several differential diagnoses, some of which could be ruled out thanks to the previous workup. These diagnoses included: CST, CCF, sino-orbital mucormycosis (SOM) or aspergillus infection, bacterial cellulitis (of sinus origin given the history of recent rhinitis), neoplasm, vasculitis and collagen vascular diseases such as granulomatosis with polyangiitis (GPA) or sarcoidosis, thyroid eye disease (TED), and finally nonspecific orbital inflammation (NSOI). We ruled out the diagnosis of CST due to normal MRI and MRV results (Fig. 1.D and Supplementary Fig. 2.A) and CCF due to the absence of a fistula on cerebral angiography (See Supplementary Fig. 2.B). Additionally, the absence of a space-occupying mass on the CT scan and MRI made a malignancy diagnosis unlikely. The absence of imaging features of myositis and normal lacrimal glands also favored against TED and sarcoidosis, respectively. Due to uncontrolled diabetes and examinations, an ENT consultation was requested to investigate SOM. During nasal endoscopy, we discovered nasal septal necrosis. A biopsy and sampling for smear and culture were performed, and a serological examination was started at the same time.

Peripheral blood tests revealed a WBC of 13,500/µL (Neutrophils: 86 %), CRP > 200 mg/L, and ESR of 99 mm/h. Negative rheumatological tests were also reported, including: P-ANCA-, C-ANCA-, anti-dsDNA-, SSA-RO-, SSB-LA-, ANA-, RF-, and ACE. Renal and urinary tests were normal, indicating there was no evidence of glomerulonephritis. Thyroid function tests were normal as well. Of course, HbA1c was 8.8. Blood cultures and PCR tests for SARS-CoV were negative. The biopsy obtained by nasal endoscopy showed evidence of infectious necrosis, and microbiology and mycology tests, including smear and lavage culture of secretions, were negative. However, the BK culture was positive for acid-AFB on Lowenstein-Jensen medium. The BK smear was 1 + for AFB with Ziehl–Neelsen stain, and PCR detected *M. tuberculosis* DNA. An HIV ELISA test was also performed on the patient, which was negative. The PPD skin test was measured at 16 mm. To complete the investigation, a sputum sample was obtained and cultured for *M. tuberculosis*, which was negative. Ziehl–Neelsen staining of the sputum was also performed and was negative. A chest CT scan was performed to investigate the presence of a pulmonary TB focus and revealed only a noncalcified nodule in the periphery of the left lung. This nodule was low-density and seemed to be more incidental than indicative of pulmonary TB. The patient refused to continue the workup for the chest CT scan finding at this stage. Given the absence of respiratory symptoms and the lack of radiologic evidence suggestive of active pulmonary tuberculosis, further molecular testing of sputum (e.g., GeneXpert) was not pursued.

Based on the histological findings and PCR confirmation, the patient was started on first-line anti-tubercular therapy (ATT) consisting of isoniazid, rifampin, pyrazinamide, and ethambutol for the initial two-month intensive phase, followed by a continuation phase with isoniazid and rifampin, in accordance with standard tuberculosis treatment guidelines [5]. Symptoms including diplopia, proptosis, and chemosis completely improved by the second month. The treatment as a two-drug regimen will continue for at least the next four months. This will depend on the response to treatment.

## Discussion

We present a rare case of isolated O-TB without pulmonary involvement in a patient with uncontrolled diabetes. The patient presented symptoms that mimicked several important diseases, including CCF, CST, and SOM. The diagnosis was ultimately made after an invasive workup. The patient lived in an area endemic for TB and had previously been referred to a tertiary ophthalmology center; however, the final diagnosis was still challenging. We do not believe that the patient was misdiagnosed, given the fact that most cases of O-TB are reported in children [5]. The diagnostic process required excluding important and potentially fatal diagnoses to reach such a difficult and rare diagnosis. However, this case review demonstrates that diseases such as O-TB can present in new ways, even to experienced clinicians, and a review of such cases is essential for ophthalmologists.

CCF can occur following trauma (high flow) or spontaneously (dural, low flow). The spontaneous type is especially prevalent in elderly women [6]. Common manifestations include proptosis, restricted movement, chemosis, corkscrew-shaped episcleral veins, increased episcleral vein pressure, and SOV engorgement as revealed by imaging [6]. Our patient had no history of trauma. Her IOP was also normal and symmetric, but the exam was consistent with other features of CCF. Therefore, she underwent cerebral angiography to confirm the diagnosis. No fistula between the carotid blood flow and the cavernous sinus was found (Supplementary Fig. 2.B).

CST is a life-threatening condition, accompanied by fever, headache, and altered state of consciousness [7]. Ptosis, ocular muscle paralysis, and disruption of the V1 and V2 nerve pathways are seen with this condition, and symptoms may progress to the opposite side due to communication between the two sinuses [7]. Our patient had no evidence of fever, headache, or altered consciousness. She had no ptosis or sensory loss in the V1 and V2 pathways. Additionally, the symptoms remained confined to the same side since the onset. MRI revealed normal intensity (Fig. 1.D) MRV did not show a flow void in the cavernous sinus (Supplementary Fig. 2.A). For this reason, CST was ruled out.

SOM was an important differential diagnosis given the patient's uncontrolled diabetes, acute presentation, and ocular congestion. However, this diagnosis seemed unlikely due to the absence of characteristic imaging findings, including bone erosion or black turbinate sign, as well as negative smears and cultures obtained from nasal endoscopy [8].

The accompanying nasal septal necrosis likely represents local infectious involvement by *M. tuberculosis*, possibly extending from the adjacent paranasal sinus. This is supported by positive AFB smear, culture, and PCR results. Even in the absence of SOV thrombosis, localized orbital inflammation can lead to venous congestion and retrobulbar fat edema, representing a vascular-mimicking presentation of orbital TB. High levels of inflammatory markers, such as leukocytosis with neutrophilia, CRP levels greater than 200 mg/dl, and ESR of 99 mm/h, support the suspicion of an acute infection or inflammatory process, such as sino-orbital fungal infection or bacterial cellulitis. However, negative initial smears and cultures, as well as confirmation of *M. tuberculosis* on additional tests, suggest that severe O-TB may also be associated with a systemic inflammatory response.

Due to the orbital inflammation and nasal septal necrosis observed during nasal endoscopy, we considered vasculitic diagnoses, such as GPA, sarcoidosis, and collagen vascular diseases, such as lupus. Rheumatological testing was performed but was negative. The patient did not have lung involvement or evidence of glomerulonephritis. This makes vasculitic and collagen vascular causes unlikely.

Despite the rarity of unilateral TED and the absence of eyelid retraction or a history of thyroid disease, we requested a TFT, which was normal. Additionally, the CT scan did not show evidence of myositis with a TED pattern.

Orbital malignancies may cause engorgement by interfering with SOV drainage; however, no evidence of a mass was seen on imaging. Additionally, the patient's response to ATT further reduces the possibility of malignancy [9].

Table 1 provides a summary of selected O-TB case reports from 2000. The cases were chosen based on having sufficient clinical, imaging, and treatment data. The majority of patients were in the pediatric and young adult age groups. The advanced age of our patient (73 years old) has never been reported before. This led us to consider a broader differential diagnosis. In most reports, a mass was visible in the eyelid or anterior orbital region in imaging results, making a direct orbital biopsy possible and therefore diagnosis easier. However, our patient's presentation was deceptive on orbital imaging; the findings were limited to SOV engorgement and retrobulbar fat enhancement. The diagnosis was made only after several invasive procedures and with the help of nasal endoscopy. As was observed in our case, none of the cases listed in the table had pulmonary TB. Our patient responded very well to treatment with ATT, as did patients in previously reported cases.

**Table 1:** Summary of reported cases of orbital tuberculosis (O-TB) ordered by age.

**Author (Year)**	**Country**	**Age (Years)/Sex**	**Presentation/Initial Impression**	**Imaging**	**Diagnosis Method**	**Outcome**
Tagoe et al. (2023) [10]	Ghana	2*/F	Upper eyelid mass with ulceration and purulent discharge/orbital rhabdomyosarcoma	CT: superolateral soft tissue with bone erosion and dural thickening	Incisional biopsy	Marked improvement after 6 months
Oliveira et al. (2004) [11]	Brazil	3, 58, 52/3 × F	Eyelid swelling/NSOI	CT: nonspecific soft tissue alterations	Incisional biopsy + immunohistochemistry	Remission in 6–9 months
Biswas et al. (2001) [12]	Bangladesh	3/M	Discharging fistula	CT: lower eyelid mass with bone erosion	Excisional biopsy + PCR for MTB	Complete regression in 6 months
Aggarwal et al. (2002) [13]	India	7/F	Painless proptosis + diplopia	CT + MRI: superolateral orbital mass with bone erosion	Surgical evacuation + tissue biopsy and AFB culture	Asymptomatic at 6 months
Gaude and Potdar (2023) [4]	India	7/F	Insidious Left upper eyelid painless swelling/Neoplasm	CT: Roof and lateral orbital wall lytic lesions	Aspiration biopsy	shrinkage in 1 month
Desai et al. (2025) [14]	India	9/F	Unilateral painless upper eyelid swelling after trauma/Hematoma	CT: Superolateral orbital lesion + bone erosion	Orbital biopsy + Culture on LJ medium + MPT 64 antigen detection assay	improvement within 2 months
Sethi et al. (2011) [15]	India	10/F	Painless infraorbital swelling	CT: Two well-marginated hypodense lesions over zygomatic & maxillary bones with erosive changes	FNA cytology, AFB positive staining + Reactive serum ELISA	Complete resolution (time not specified)
Dewan et al. (2006) [16]	India	14/F	Progressive vision loss + proptosis/optic nerve glioma	MRI: Intraorbital mass with cranial extension	Excisional biopsy	Improved vision from NLP to count finger 1 m
Kaur and Agrawal (2005) [17]	India	17/M	Painless orbital mass/Orbital Pseudo-tumor** → Discharging sinusitis and cervical lymphadenitis/suspected S. aureus orbital abscess	CT: soft lesion in left lateral rectus tendon	FNA cytology, ELISA anti-MTB IgM	complete recovery at 6 months
Yoon et al. (2019) [18]	Korea	18/F	Lower eyelid nodule/abscess	CT: Small abscess cavity with no bone erosion	Excisional biopsy	Recovery after 6 months
Shome et al. (2005) [19]	India	60/F	Enophthalmus + Hard mass in the lower orbit/Orbital metastasis (scirrhous carcinoma) or Sclerosing NSOI	CT: anterior infratemporal mass with no bone erosion	Incisional biopsy + PCR	Favorable Recovery (time not specified)

LJ medium: Lowenstein-Jensen medium, MTB: Mycobacterium tuberculosis.

* Exact age is not specified in the manuscript.

** Patient was initially treated with systemic corticosteroid which resulted in purulent discharge.

This report has several strengths. First, our patient is the oldest reported case of O-TB in the literature with an atypical presentation that mimicked vascular causes. The validity of this report is demonstrated by comprehensive multimodal imaging, microbiological confirmation by smear, culture, and PCR, as well as the patient's clinical response to ﹶATT. However, due to the nature of a single case report, there are several limitations. The absence of histopathological sampling from the orbit itself and the absence of molecular testing of sputum limit the generalizability of our findings. Nevertheless, this report provides important clinical insights into the atypical presentation of O-TB that could help prevent delayed diagnoses in similar cases.

## Conclusion

This case highlights that O-TB can rarely present with vascular imaging findings, such as SOV engorgement, which can lead to an initial suspicion of CST. Recognizing this unusual presentation can prevent mismanagement, especially in endemic areas. O-TB should be considered in the differential diagnosis of orbital inflammation with imaging findings mimicking vascular emergencies, especially when routine vascular work-ups are inconclusive.

## Abbreviations

O-TB: Orbital Tuberculosis

MTB: *Mycobacterium tuberculosis*

TB: Tuberculosis

SOV: Superior Ophthalmic Vein

CCF: Carotid-Cavernous Fistula

CST: Cavernous Sinus Thrombosis

MRI: Magnetic Resonance Imaging

MRV: Magnetic Resonance Venography

PCR: Polymerase Chain Reaction

AFB: Acid-Fast Bacilli

ATT: Anti-Tuberculosis Treatment

SOM: Sino-Orbital Mucormycosis

GPA: Granulomatosis with Polyangiitis

TED: Thyroid Eye Disease

NSOI: Nonspecific Orbital Inflammation

BCVA: Best-Corrected Visual Acuity

IOP: Intraocular Pressure

DKA: Diabetic Ketoacidosis

LJ medium: Lowenstein-Jensen medium

HIV: Human Immunodeficiency Virus

PPD: Purified Protein Derivative

## Authorship

All authors attest that they meet the current ICMJE criteria for Authorship.

## CRediT authorship contribution statement

**Mohsen Zare:** Writing – review & editing, Writing – original draft, Investigation. **Mojdehi Amirhossein:** Writing – review & editing, Investigation, Conceptualization. **Mansoor Shahriari:** Validation, Supervision, Project administration, Conceptualization.

## Ethical approval

This article is a single case report. Written informed consent was obtained from the patient for publication of clinical information and images.

## Consent

Consent to publish this case report has been obtained from the patient in writing. This report does not contain any personal identifying information.

## Financial Support

None.

## Meeting Presentation

None.

## Funding

No funding or grant support.

## Conflict of Interest

No conflicting relationship exists for any author.

## Conflicts of Interest

The authors have no financial disclosures.

## Declaration of Competing Interest

The authors declare that they have no known competing financial interests or personal relationships that could have appeared to influence the work reported in this paper.

## Appendix A Supplementary material

Supplementary Fig. 1**.** (A) Axial and (B) coronal T1-weighted post-contrast fat-suppressed MRI images showing multiple foci of retrobulbar fat enhancement (arrows) in the left orbit, consistent with orbital inflammation.

Supplementary Fig. 2. (A) Sagittal MR venography demonstrating patent cavernous sinuses bilaterally, with no evidence of thrombosis. (B) Sagittal cerebral angiography showing no evidence of carotid-cavernous fistula. The ophthalmic artery and cavernous segment of the internal carotid artery are labeled.

## References

[1] Sallam A., Karimaghaei S., Neuhouser A.J., Tripathy K. (2024). Ocular Tuberculosis. Integr Sci.

[2] Hayward S.E. (2021). Extrapulmonary tuberculosis among migrants in Europe, 1995–2017. Clin Microbiol Infect.

[3] Madge S.N. (2008). Orbital tuberculosis: a review of the literature. Orbit.

[4] Gaude P., Potdar N. (2023). Orbital tuberculosis. Indian J Ophthalmol Case Rep.

[5] Sulaiman I.I. (2024). Challenges and insights in the diagnosis and management of orbital tuberculosis: a systematic review of 113 cases. Cureus.

[6] Kohli G.S., Patel B.C. (2023). Carotid cavernous fistula. Neurol Neurosurg Emerg.

[7] Mira F., Costa B., Paiva C., Andrês R., Loureiro A. (2014). Cavernous sinus thrombosis. Rev Bras Oftalmol.

[8] Safder S., Carpenter J.S., Roberts T.D., Bailey N. (2010). The ‘black turbinate’ sign: an early MR imaging finding of nasal mucormycosis. AJNR Am J Neuroradiol.

[9] Adam C.R. (2018). Dilated superior ophthalmic vein: clinical and radiographic features of 113 cases. Ophthal Plast Reconstr Surg.

[10] Tagoe L.G. (2023). Orbital tuberculosis mimicking an ocular malignancy: a case report. Heal Sci Investig J.

[11] Oliveira B.F.T. (2004). Orbital tuberculosis diagnosed by immunohistochemistry: case reports. Rev Inst Med Trop Sao Paulo.

[12] Biswas J., Chowdhury B.R., Kumar S.K., Therese K.L., Madhavan H.N. (2001). Detection of Mycobacterium tuberculosis by polymerase chain reaction in a case of orbital tuberculosis. Orbit.

[13] Aggarwal D., Suri A., Mahapatra A.K. (2002). Orbital tuberculosis with abscess. J Neuroophthalmol.

[14] Desai A., Rai M., Madiwale C. (2025). Unveiling the hidden culprit: a case report on orbital tuberculosis. J Med Sci Health.

[15] Sethi A., Agarwal A.K., Girhotra M., Naithani P. (2011). Tuberculosis: an extremely unusual cause of orbital wall erosion. Orbit.

[16] Dewan T., Sangal K., Premsagar I.C., Vashishth S. (2006). Orbital tuberculoma extending into the cranium. Ophthalmologica.

[17] Kaur A., Agrawal A. (2005). Orbital tuberculosis - an interesting case report. Int Ophthalmol.

[18] Yoon H.S., Na Y.C., Lee H.M. (2019). Primary orbital tuberculosis on the lower eyelid with cold abscess. Arch Craniofac Surg.

[19] Shome D., Honavar S.G., Vemuganti G.K., Joseph J. (2006). Orbital tuberculosis manifesting with enophthalmos and causing a diagnostic dilemma. Ophthal Plast Reconstr Surg.

