# Supplementary figures and images for: Orbital tuberculosis mimicking a vascular emergency: A case report of superior ophthalmic vein engorgement
Source: IDCases. 2026 Feb 22;43:e02534. doi: 10.1016/j.idcr.2026.e02534 (PMC12969041; doi:10.1016/j.idcr.2026.e02534)

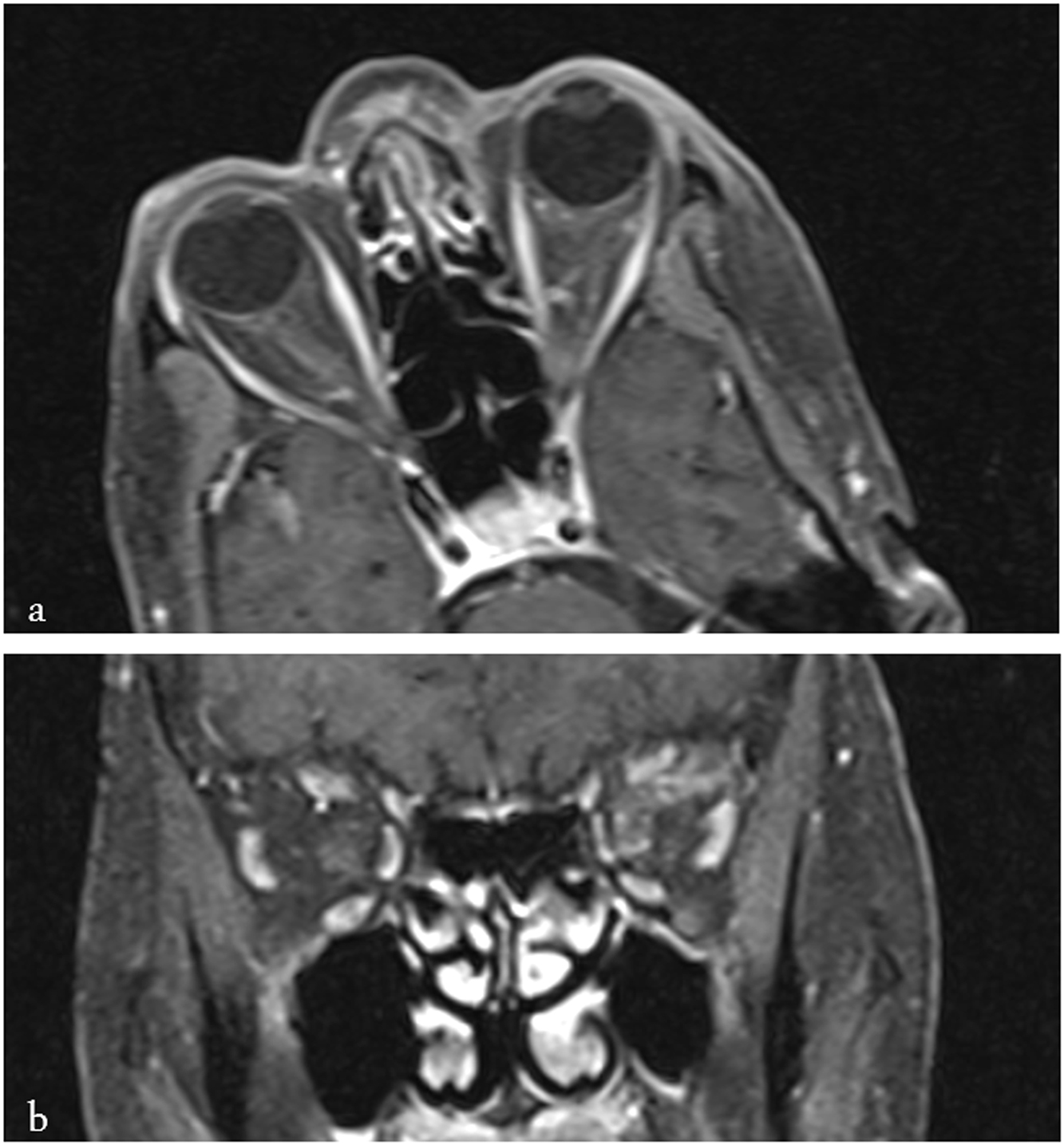

Supplement: Supplementary file 1 — Supplementary Fig. 1. (A) Axial and (B) coronal T1-weighted post-contrast fat-suppressed MRI images showing multiple foci of retrobulbar fat enhancement (arrows) in the left orbit, consistent with orbital inflammation. [file mmc1.jpg]

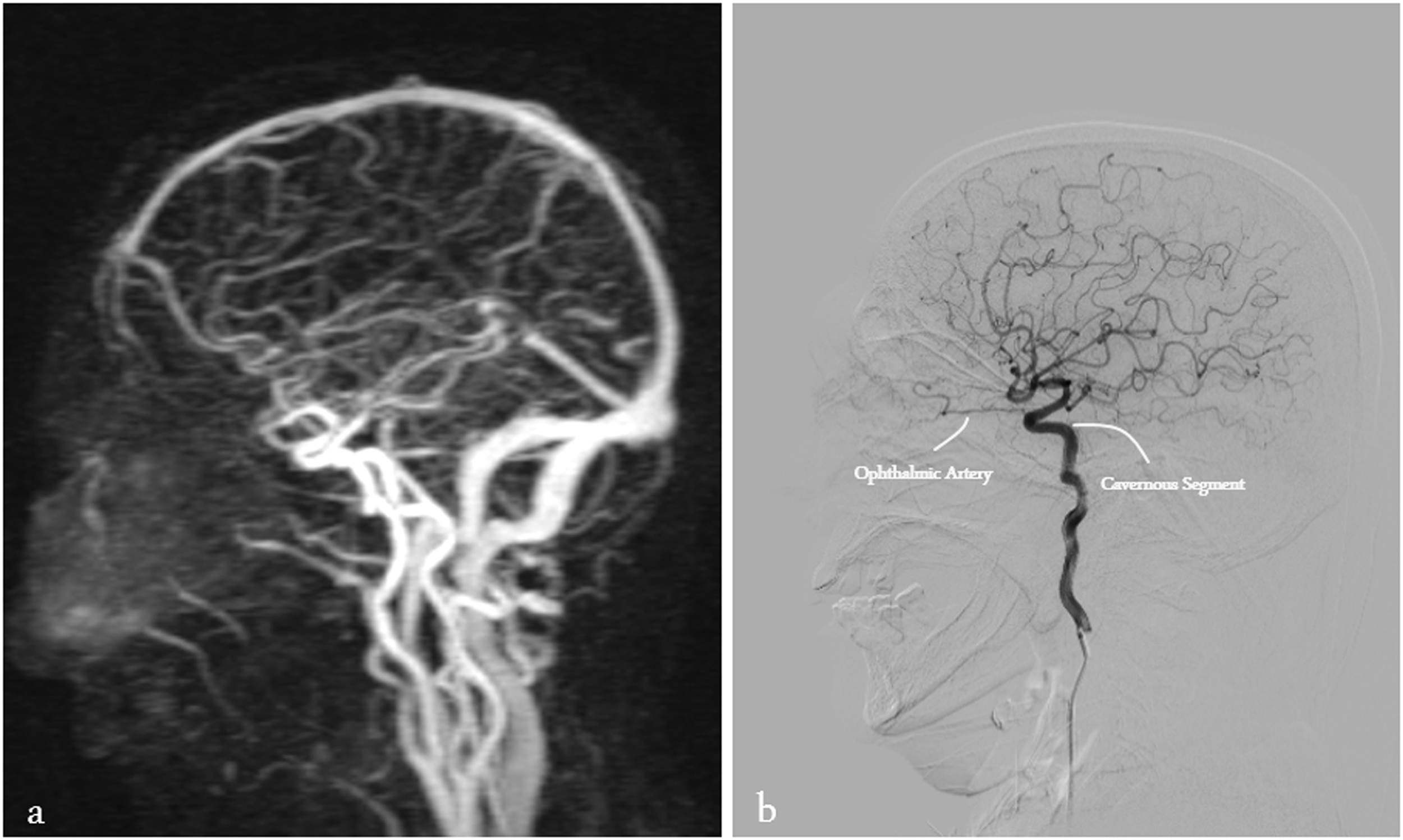

Supplement: Supplementary file 2 — Supplementary Fig. 2. (A) Sagittal MR venography demonstrating patent cavernous sinuses bilaterally, with no evidence of thrombosis. (B) Sagittal cerebral angiography showing no evidence of carotid-cavernous fistula. The ophthalmic artery and cavernous segment of the internal carotid artery are labeled. [file mmc2.jpg]
